# Ruptured Multifocal Hepatic Aneurysms in a Woman with Systemic Lupus Erythematosus Successfully Treated with Transcatheter Arterial Embolization: A Case Report and Literature Review

**DOI:** 10.1155/2019/6272419

**Published:** 2019-02-26

**Authors:** Sz-Iuan Shiu, Su Ann Yong, Kuo-Lung Lai, Chih-Wei Tseng, Chen-Yu Wang, Bor-Jen Lee, Yi-Hsing Chen

**Affiliations:** ^1^Division of Gastroenterology and Hepatology, Department of Internal Medicine, Taichung Veterans General Hospital, Taichung, Taiwan; ^2^Department of Critical Care Medicine, Taichung Veterans General Hospital, Taichung, Taiwan; ^3^China Medical University, Taichung, Taiwan; ^4^Division of Allergy, Immunology and Rheumatology, Department of Internal Medicine, Taichung Veterans General Hospital, Taichung, Taiwan; ^5^Faculty of Medicine, National Yang-Ming University, Taipei, Taiwan

## Abstract

To present a first reported case of ruptured multifocal hepatic aneurysms in a woman with systemic lupus erythematosus (SLE) who was treated successfully with transcatheter arterial embolization (TAE) in literature, similar cases in the previous English literature were also reviewed and analyzed to summarize the clinical manifestations, management, and outcome in these patients. The data were gathered from the medical record and literature reviews were searched from PudMed. In our review, patients with SLE-related hepatic aneurysms were often middle-aged females. Most of them presented with acute abdominal pain and hypotension. The overall mortality rate was 50%, but it was lower (12.5%) in patients who received TAE. Both TAE and surgical intervention are used to treat SLE-related hepatic aneurysms. Our review raised concerns about early detection, diagnosis, and prompt intervention of possible hepatic aneurysm rupture in patients with SLE.

## 1. Introduction

Hepatic aneurysms are rare with an estimated incidence of approximately 0.002%. Very few cases have been reported in patients with systemic lupus erythematosus (SLE) [1] and the pathological mechanism, optimal treatment, and prognosis in this patient population are not clear. We present a case of multifocal hepatic aneurysm rupture in a woman with SLE successfully treated with transcatheter arterial embolization (TAE).

## 2. Case Presentation

A 35-year-old woman presented to the emergency department (ED) complaining of sudden-onset, persistent, moderately severe, left-sided headache with focal left visual field defect followed by right limb clumsiness three hours priorly. She was diagnosed with SLE 11 years previously after developing nephritis, intermittent arthritis, thrombocytopenia, and chronic leg ulcers. She was lupus anticoagulant positive. Treated with monthly cyclophosphamide pulse therapy followed by trimonthly injections for the first two and a half years, she remained free of flares on daily maintenance therapy (azathioprine, 50 mg; hydroxychloroquine, 200 mg; prednisolone, 5 mg (0.05 mg/kg/day); and aspirin, 100 mg).

Neurological exam revealed right homonymous hemianopsia without facial palsy but with right-sided hyperesthesia and dysmetria. Brain computed tomography (CT) and magnetic resonance angiography showed acute cerebral infarction in the left posterior cerebral artery territory involving the thalamus and occipital lobe complicated by minimal left temporo-occipital subarachnoid hemorrhage. She was weakly positive for lupus anticoagulant and borderline positive for anticardiolipin antibodies.

**Figure 1:**
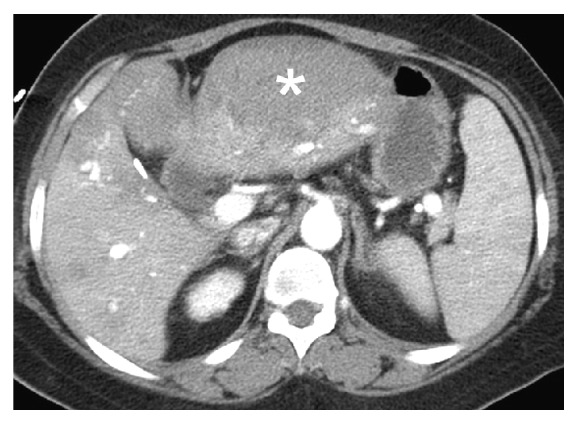
The computed tomographic angiography of abdomen of first time revealed subcapsular hematoma (asterisk) with contrast extravasation at left lateral segment of liver.

**Figure 2:**
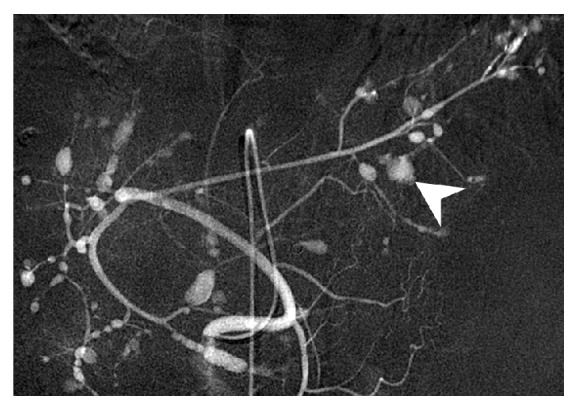
The angiography of abdomen revealed diffuse hepatic artery aneurysms over bilateral liver parenchyma with contrast extravasation over branches of left hepatic artery (arrowhead).

**Figure 3:**
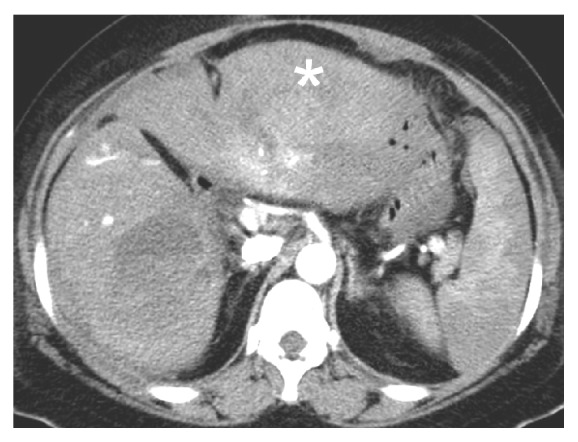
The computed tomographic angiography of abdomen 3 months later showed complete resolution of subscapular hematoma (asterisk). A residual hematoma over right lobe of liver was noted but without any clinical manifestation.

Her symptoms gradually improved, but she developed sudden-onset, severe epigastric pain with tachycardia, hypotension, and altered level of consciousness two weeks after hospitalization. Her hemoglobin dropped from 114 to 88 g/L, and abdominal CT angiography (CTA) showed a massive subcapsular hematoma with contrast extravasation in the left lateral segment of the liver (Figure 1). Emergent angiography showed diffuse hepatic artery aneurysms bilaterally over the liver parenchyma with contrast extravasation from a left hepatic subcapsular hematoma (Figure 2). Diagnosed with hepatic aneurysm rupture, embolization of left proximal hepatic artery with Gelfoam cubes was performed. However, her tachycardia persisted, and her hemoglobin was 66 g/L the next day. Follow-up CTA showed a new hematoma over liver segment 7/8. A second superselective TAE with Gelfoam cubes was performed via branches of right hepatic artery after which her vital signs stabilized. Concerned that antiphospholipid antibodies were responsible for the vascular events, rituximab was administered (500 mg in two consecutive doses two weeks apart). Follow-up abdominal CTA three months later showed resolution of the hepatic aneurysms (Figure 3).

## 3. Discussion

Hepatic aneurysm rupture is rare but life-threatening. SLE-related hepatic aneurysms are even rarer, with only 13 reported cases in the literature [1–8]. Mean patient age at admission was approximately 34 years with a female predominance (71%) (Table 1). Mean SLE duration was 7.1 years with three-fourths of patients using continuous steroids and only 7% being hypertensive. Most patients presented with acute abdominal pain (78.6%) and hypotension (57.1%). The hepatic aneurysms were on the right lobe in 50%, left lobe in 41.6%, and bilateral lobes in 8.4%. Approximately 83% of patients had a ruptured aneurysm at presentation. The overall mortality rate was 50%, but it was lower (12.5%) in patients who received TAE. Lobectomy may be required as rescue therapy in 21.4% of patients.

The precise mechanism of hepatic aneurysm in SLE is not yet clear. Several hypotheses have been proposed including collagen degeneration, and destruction of smooth muscle and elastic fibers due to vasculitis as well as long-term steroid use [8]. Kurata et al. proposed two different pathogenic mechanisms for aortic aneurysms in lupus patients [9]. In younger patients, systemic vasculitis causes cystic medial degeneration leading to aortic dissection. In older patients on long-term glucocorticoid therapy, aneurysm formation is more likely to be caused by atherosclerosis due to aging. Our patient was relatively young, and the aneurysms resolved with treatment, so vasculitis is more likely to be the cause.

Owing to the large proportion of patients presenting with rupture as the first and only manifestation of hepatic aneurysm, the mortality rate is high, so rapid, appropriate diagnosis and management are crucial. We recommend CTA for diagnosis. Both TAE and surgical intervention are used to treat hepatic aneurysms [1, 10–13]. Choice of treatment modality depends on patient vital signs, aneurysm location, and presence of hepatic collateral circulation. TAE is preferred in patients with a patent portal vein who do not have obstructive jaundice but are a high surgical risk. Surgical intervention with vascular reconstruction is preferred in patients with obstructive jaundice and profound hypovolemic shock despite rapid resuscitation. In our case, prophylactic embolization of the main hepatic artery was performed during the second TAE to reduce intra-aneurysmal pressure, which may have contributed to shrinkage and disappearance of the aneurysms found at her three-month follow-up visit.

B cell depletion with the anti-CD20 monoclonal rituximab has been found to be effective in patients with SLE in several open-label cohort studies [14] and rituximab has also been suggested in refractory SLE [15]. Under the concern that hepatic aneurysm might be related to refractory SLE, we prescribed rituximab as induction and maintenance therapy for residual hepatic aneurysm after embolization. No residual hepatic aneurysm was discovered at her three-month follow-up visit.

To our knowledge, this is the first reported case of bilateral multifocal hepatic aneurysm rupture in a young woman with SLE successfully treated with TAE. It emphasizes the need for rapid diagnosis and treatment to achieve a successful clinical outcome in patients with hepatic artery aneurysm.

## Figures and Tables

**Table 1:** The demographic data of patients with SLE-related hepatic aneurysms rupture in literature review.

Patient (reference)	Age	Gender	CAD risk*∗*	Duration since SLE diagnosed (years)	Steroid use prior to admission	Presentation	Diagnostic tools	Hepatic aneurysms location	Hepatic rupture	Intervention	Outcome
Paronetto F et al., 1964	26	F	No	6	Yes	Abd pain and hypotension	Autopsy	NA	NA	Conservative therapy with transfusion	Death
Haslock I, 1974	32	F	No	14	Yes	Abd pain and hypotension	Laparotomy	Right lobe	Yes	Lobectomy	Death
Levitin PM et al., 1977	27	F	No	> 3	No	Abd pain	Laparotomy	NA	NA	Liver packing	Death
McCollum CN et al., 1979	33	F	HTN	12	Yes	Abd Pain and hypotension	Laparotomy	Left lobe	Yes	Left lateral lobectomy	Death
Trambert J et al., 1989	49	M	No	< 1	No	Abd pain	Abd CT and angiography	Left lobe	Yes	Embolization	Survival
Stratton R et al., 1999	24	F	No	3	Yes	Abd pain	Abd CT	Left lobe	No	Embolization, pulse therapy of steroid, and cyclophosphamide monthly	Survival
Huang JW et al., 1999	31	F	No	10	Yes	Hematemesis and tarry stool	Abd CT	Right lobe	Yes	Embolization	Death
Kong KO et al., 2002	21	M	No	2	Yes	Abd pain and cardiac arrest	Autopsy	Right lobe	Yes	Cardiopulmonary resuscitation	Death
Singh R et al., 2003	33	F	No	10	Yes	Abd pain and hypotension	Laparotomy	Right lobe	Yes	Laparotomy and cauterization of bleeding vessels	Death
Yamazaki K, 2004	34	M	No	13	Yes	Abd pain and hypotension	Abd CT	Left lobe	Yes	Embolization	Survival
Pollono EN et al., 2010	56	F	No	0.5	No	GI bleeding	Abd CT and angiography	Right lobe	No	Embolization	Survival
Liu C et al., 2011	31	M	No	7	Yes	Hemobilia	Angiography	Left lobe	Yes	Embolization and left lateral lobectomy	Survival
Reiter DA et al., 2013	41	F	No	NA	NA	Abd Pain and hypotension	Abd CT	Right lobe	Yes	Embolization	Survival
Our patient	35	F	No	11	Yes	Abd pain, hemobilia, and hypotension	Abd CT and angiography	Bilateral lobes	Yes	Embolization	Survival
Mean	33.8	4M10F	7%	7.1 yrs [1–14]	76.9%	78.6% abd pain; 14.3% hemobilia; 57.1% with hypotension; 28.6% with GI bleeding (including hemobilia)		50% right lobe; 41.6% left lobe; 8.4% bilateral lobe	83.3%	57.1% with embolization (mortality rate 12.5% within patients with embolization); 21.4% with lobectomy (mortality rate 67% within patients with lobectomy);	50% mortality

*∗*HTN, hyperlipidemia, DM, PAOD, stroke.

F: female, M: male, NA: not available, HTN: hypertension, Abd: abdominal, CT: computed tomography, and GI: gastrointestinal.
